# Synthesis of Silicon and Germanium Oxide Nanostructures via Photonic Curing; a Facile Approach to Scale Up Fabrication

**DOI:** 10.1002/open.202300260

**Published:** 2024-02-02

**Authors:** Najma Khatoon, Binod Subedi, Douglas B. Chrisey

**Affiliations:** ^1^ Department of Physics and Engineering Physics Tulane University New Orleans LA 70118

**Keywords:** germanium oxide, nucleation and growth, photonic curing, roll-to-roll manufacturing, silicon oxide

## Abstract

Silicon and Germanium oxide (SiO_x_ and GeO_x_) nanostructures are promising materials for energy storage applications due to their potentially high energy density, large lithiation capacity (~10X carbon), low toxicity, low cost, and high thermal stability. This work reports a unique approach to achieving controlled synthesis of SiO_x_ and GeO_x_ nanostructures via photonic curing. Unlike conventional methods like rapid thermal annealing, quenching during pulsed photonic curing occurs rapidly (sub‐millisecond), allowing the trapping of metastable states to form unique phases and nanostructures. We explored the possible underlying mechanism of photonic curing by incorporating laws of photophysics, photochemistry, and simulated temperature profile of thin film. The results show that photonic curing of spray coated 0.1 M molarity Si and Ge Acetyl Acetate precursor solution, at total fluence 80 J cm^−2^ can yield GeO_x_ and SiO_x_ nanostructures. The as‐synthesized nanostructures are ester functionalized due to photoinitiated chemical reactions in thin film during photonic curing. Results also showed that nanoparticle size changes from ~48 nm to ~11 nm if overall fluence is increased by increasing the number of pulses. These results are an important contribution towards large‐scale synthesis of the Ge and Si oxide nanostructured materials which is necessary for next‐generation energy storage devices.

## Graphical abstract

Silicon oxide (SiO_x_) and germanium oxide (GeO_x_) nanoparticles are promising candidates for energy storage applications. We synthesized SiO_x_ and GeO_x_ nanostructures by employing photonic curing; a low‐cost roll‐to‐roll instantaneous process. This work is a step to optimize photonic curing for semiconductor oxide nanostructures synthesis on a large scale with nanometric control for next generation energy applications.

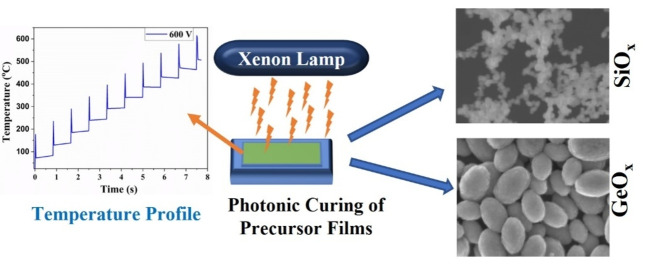

## Introduction

Tunable and unique properties of nanoparticles as compared to their bulk material properties make them highly interesting for many applications ranging from biology and energy applications to electronic applications.^[1]^ Their unique properties include high surface‐to‐volume ratio, chemical, electrical, magnetic, and optical properties.[^2^, ^3^, ^4^, ^5^] Moreover, these properties can be changed by controlling the size, shape, and phase of nanoparticles.[^3^, ^4^, ^5^, ^6^, ^7^, ^8^, ^9^, ^10^, ^11^, ^12^, ^13^, ^14^] Table 1 summarizes size‐dependent properties and applications of nanoparticles. Semiconductor nanoparticles are particularly interesting owing to their unique optical and electronic properties, and other disparate applications.[^15^, ^16^, ^17^] Si and Ge nanoparticles can be used as anode material for Li−Ion batteries, as current leakage prevention in metal‐oxide‐semiconductor (MOS) memory devices, and as improving photostability of material.[^17^, ^18^, ^19^]

**Table 1:** Summary of nanomaterial′s size‐dependent applications and properties. Based on size nanoparticles are categorized into four types with example nanoparticles, their properties, and applications.

Dimension of Nanomaterials	Nanomaterials	Properties	Applications	References
Zero dimensional (0D)	Quantum dots	Optical stability	Nanomedicine	[20–23]
xyz <100 nm	Fullerenes	Photoluminescence	Cosmetics	
	Nanoclusters	Chemical inert	Bioelectronics	
	Clusters	Biocompatible	Biosensor	
	Graphene QD		Biochip	
One dimensional (1D)	Nanobars	Chemically pure	Photochemical	[23,24]
xy <100 nm	Nanowires	Metallic	Photovoltaic	
	Carbo nanotubes	Ceramics	Energy storage devices	
	Nanoribbons	Polymers		
		Single crystalline		
Two dimensional (2D)	Nanofilms	Large specific area	Catalysis	[25–31]
x,y or z <100 nm	Nanolayers	High electrical and thermal conductivity	Optoelectronic	
	Graphene		Energy storage	
			Biomedicine	
			Sensors	
			Supercapacitors	
			Batteries	
Three dimensional (3D)	Polycrystals	Ultra‐stable	Batteries Nanoelectrodes	[23,32]
xyz >100 nm	Diamonds		Water splitting	
	Graphene oxides		Photonics	
	Aerogels		Energy Conversion	

Column 14 (IVA) nanoparticles like C, Si, and Ge, are promising semiconductor nanoparticles due to their high natural abundance, unique optical properties, surface chemistry, and low toxicity.^[33]^ Si nanoparticles opened a new era to their applications in electronics, sensors, biomedical and energy storage due to its distinguished physical and chemical properties.[^34^, ^35^, ^36^, ^37^, ^38^, ^39^] Si has an indirect bandgap, and it shows promising quantum confinement for a smaller Bohr radius (~4 nm).^[34]^ Silicon is a promising candidate for lithium‐ion batteries due to its high theoretical capacity ~3580 mAhg^−1^. Recently, Koki et. al. reported low temperature nanostructured silicon films for lithium‐ion batteries with enhanced capacity of 3200 mAhg^−1^ at 0.1 C rate amenable to flexible anodes for rechargeable batteries.^[40]^ Ge‐based nanoparticles also possess interesting properties, such as high refractive index, narrow band gap, high ion intercalation capacity, low toxicity, and high charge carrier mobilities.^[41]^ Ge nanoparticles gained attention in the research area because of their wide range of applications in energy storage, bioimaging and optoelectronics etc.^[42]^ Unlike most of other semiconductor nanoparticles, Ge nanoparticles show quantum confinement effects at relatively larger particle sizes due to their small bandgap^[43]^ and large Bohr radius.^[44]^ Ge nanoparticles are low‐cost^[45]^ and comparatively more environmentally friendly semiconductor nanoparticles.^[46]^ These properties along with favorable electronic properties^[47]^ of Ge nanoparticles makes them promising candidate for energy storage and conversion devices like processing CuInSe_2_ for solar cells, field effect transistors, processing nanostructured TiO_2_ films, soldering on poly(ethyleneterephthalate) and polyimide foils, recrystallization of amorphous silicon, and conductivity enhancement of graphene inks, photodetectors as well as for biological imaging and flash memory devices.[^43^, ^45^, ^46^, ^48^, ^49^, ^50^, ^51^, ^52^, ^53^] Table 2 summarizes the properties and applications of Si and Ge nanoparticles.

**Table 2:** Summary of properties, applications, and synthesis routes for Si and Ge nanoparticles. The table shows possible similar applications of both Ge and Si nanoparticles along with the synthesis routes and references.

	Properties	Applications	Synthesis	References
Silicon Nanoparticles	Low cost	Quantum computing Sensors	Sol‐gel processing	[22], [54–60], [61–67], [1, 15, 67]
	Size dependent optical and electrical properties	Catalytic applications Bioimaging	Chemical vapor deposition	
	Indirect bandgap High surface to volume ratio	Construction materials Drug delivery system	Spray pyrolysis	
		Energy storage devices	Microemulsion Thermal evaporation, sputtering	
		Calorimetric diagnostics	Milling	
		Photothermal therapy	Laser ablation	
			Plasma techniques	
Germanium Nanoparticles	Low toxicity		Etching	[68], [69–73], [61–67], [1, 15, 67]
	Size dependent optical and electrical properties			
	Small bandgap			
	Photo‐luminous			
	Crystallinity			

Synthesis methods for semiconductor nanoparticles can be classified into two primary categories: solution‐phase chemical methods and vapor‐based techniques.^[15]^ Techniques available to synthesize semiconductor nanoparticles include sol‐gel processing, chemical vapor deposition, spray pyrolysis, microemulsion,^[74]^ condensation, thermal evaporation, sputtering, and milling.^[1]^

Research endeavors in the field of nanoparticle investigation are primarily focused on developing synthesis techniques that allows precise control over the size, composition, surface chemistry, and morphology of nanoparticles.^[41]^ Microwave annealing, photochemical activation, and flashlamp‐based pulsed‐light processes are among most popular methods to process oxide semiconductors.[^75^, ^76^, ^77^, ^78^, ^79^] Flashlamp‐based processes are also known as photonic curing. Photonic curing is a versatile processing method that offers to instantaneously process films at high temperatures. In photonic curing, heating intervals are very short (~1–2 ms) which results in nonequilibrium heating and creates a thermal gradient from the top of the thin film to the bottom of the substrate. With the help of integrated SimPulse software, the temperature profile of the material and substrate at different thicknesses can be estimated for varying curing conditions.[^75^, ^80^] This temperature profile as a function of time further allows us to optimize the processing parameters to obtain materials with desired characteristics.

Photonic curing is a low bulk thermal exposure sintering method developed by NovaCentrix (Austin, TX) in 2006.^[81]^ It uses a xenon flashlamp to produce high‐intensity pulsed light, which can cure materials on low‐temperature substrates without damaging the substrates. This technique is novel due to its ability to thermally process materials to temperatures approaching 1,200 °C on low temperature substrates that would decompose at temperatures greater than 400 °C. The pulse waveform, amplitude, duration, number, and frequency can be changed by the pulse editor option in PulseForge. This novel technique allows the high‐intensity light on a wide area, cures the material in a fraction of a second, and thus is amenable for the roll‐to‐roll industrial‐scale method.^[80]^ Based on its high speed, photonic curing can be used to process large areas (~200 cm^2^ per 10 cm long xenon flash lamp) in less than 2 ms for nanomaterial synthesis.^[82]^ Photonic curing is rapidly gaining attention as a separate method to process material as well as in assisting various fabrication techniques. For example, sintering and functionalization of nanoparticles, processing of high‐temperature films on low‐temperature substrates,^[83]^ processing of grids for organic cell applications,^[84]^ synthesis of dielectrics for organic field‐effect transistors,^[85]^ fabrication of metal oxide thin film transistors,[^86^, ^87^] processing of oxide electronics and flexible electronics^[88]^ have been achieved by using photonic curing. Photonic curing has been employed for the sintering of nanoparticles by heating the nanoparticles to the extent that they diffuse to form thin films.^[89]^ Herein we report the photonic curing for one‐step synthesis of Si and Ge oxide nanoparticles that also provides a facile approach to scale‐up.

**Figure 1:**
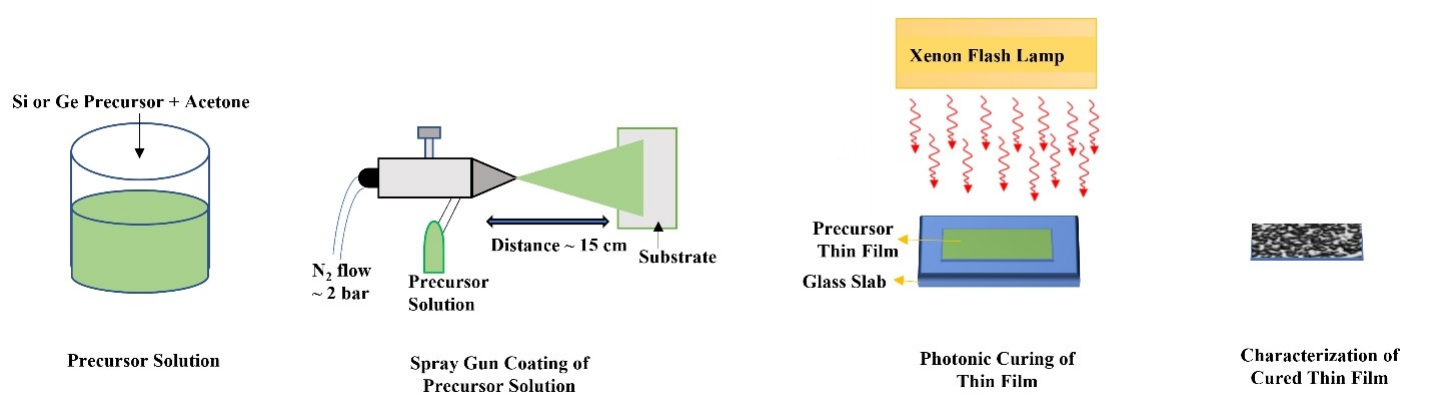
Schematic illustration of photonic curing process using PulseForge 1300. Nitrogen (N_2_) with 2 bar pressure is used to spray precursor solution on a glass substrate. The thin films are then processed via photonic curing and then characterized.

The schematic illustration of the processing of thin film via photonic curing is shown in Figure 1. The schematics show that precursor solution is sprayed on a substrate followed by photonic curing and then characterization. To check the effect of PulseForge parameters on the synthesis of nanoparticles, overall fluence was varied (from 80 Jcm^−2^ to 160 Jcm^−2^) by changing the number of pulses from 10 to 20. This whole process takes less than 30 minutes (including thin film preparation). During photonic curing, the precursor undergoes photochemical and thermal processes followed by rapid quenching which changes the structure and morphology of the material in thin films. The silicon and germanium‐based nanostructures synthesized in this work are oxidized on the surface, hence named silicon and germanium oxide (SiO_x_ and GeO_x_) nanostructures.

## Results and Discussion

**Figure 2:**
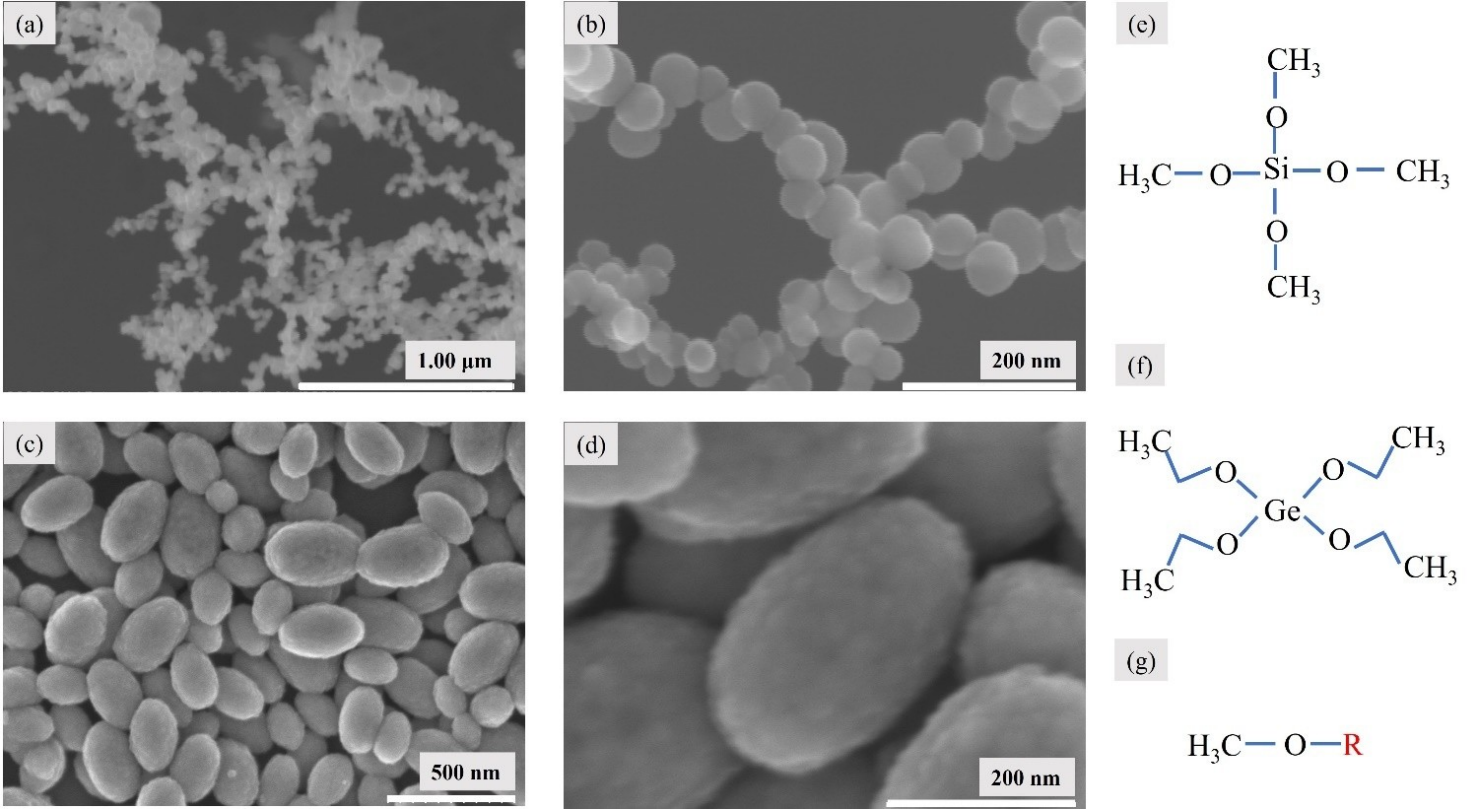
(a & b) SEM images at high and low‐resolution for Si precursor 0.1 M, treated at 600 V for 10 pulses, (c&d) at high and low‐resolution SEM images for Ge precursor 0.1 M photonically processed at 600 V for 10 pulses resulting in a total fluence of 80 Jcm^−2^. (e–f) molecular structure for Si, Ge precursor, and (g) is the molecular structure for mono and tri substitutes for the precursors.

Scanning electron microscopy (SEM) (Hitachi S‐4800, Hitachi Corp., Tokyo, Japan) was employed for the nanostructure characterization of photonically cured thin films. Figure 2 shows the SEM results of as‐prepared thin films after photonic curing. Figure 2(a & b) and Figure 2(c & d) show nanoparticles synthesized by curing Si and Ge precursor thin films respectively. SEM images show the spherical morphology of Si oxide nanoparticles connected in the form of a chain (Figure 2a & b). Ge oxide nanoparticles are oval‐shaped with scale‐like morphology on the surface as shown in Figure 2(c & d).

To explain the synthesis of these nanoparticles, it is vital to understand the mechanism of photonic curing. In the process of treating thin films with light from the xenon flashlamp (PulseForge), high‐intensity pulsed light is focused into thin film on low‐temperature substrates in a very short time (~milliseconds). This intense pulsed light from xenon flash lamp is absorbed by the thin film and eventually released as heat. The absorbed energy initiates^[89]^ the photochemical reactions in thin film which leads to nanomaterial synthesis. The nanomaterials synthesis process begins with bond breaking once photochemical reactions are initiated. Figure 2(e & f) shows the presence of Si−O and Ge−O bonds in the molecular structure for Si and Ge precursor respectively along with the O−CH_3_ bond. The bond dissociation energy for Si−O (oxygen silicon bond) is 99.6 kJmol^−1^±13.4 kJmol^−1^ and 657.5 kJmol^−1^±13.4 kJmol^−1^ for Ge−O (oxygen germanium bond).^[90]^ The bond dissociation energy for O−CH_3_ is 246.8, 232.2 and 217.9 kJmol^−1^ for mono, di, and tri substituents respectively. The molecular structure for mono, di or tri substituent is shown in Figure 2(f) which is a methoxy functional group and will undergo photochemical reactions initiated due to energy absorbed by molecules. The O−CH_3_ bond dissociation enthalpy corresponds to the enthalpy change in the following reaction (gas phase),^[91]^

(1) $$RO-CH_{3}\left(g\right)\to RO^{.}\left(g\right)+CH_{3}^{.}\left(g\right)$$

From Figure (e–g) we can see that the above‐said bonds are present in the precursors. RO and CH_3_ are alkoxy and methyl radicals respectively. These radicals will take part in transient intermediate chemical reactions that lead to the formation of SiO_x_ or GeO_x_ nanoparticles. The nanoparticles will be oxide (silicon oxide and germanium oxide) nanoparticles due to the presence of oxygen as the processing of thin films takes place under ambient conditions.

It is also vital to understand the mechanism of heat conduction during photonic curing. Heat conduction during photonic curing can be explained by using a 1‐D conduction equation,

(2) $$\rho c_{P}\frac{\partial T}{\partial t}=\frac{\partial }{\partial x}\left(k\frac{\partial T}{\partial x}\right)+S$$

Where $\rho ,\;\;c_{P},\;k$ and S presents density, specific heat at constant temperature, thermal conductivity, and volumetric source term respectively. This equation is subject to time‐dependent heat flux boundary conditions. The heat pulse from photonic curing can be treated as surface heat flux or volumetric source.^[80]^ In this case, we consider that photonic curing is volumetric heat flux. In the case of volumetric heat flux, equation 2 gives,

(3) $$\frac{q^{"}}{q_{0}^{"}}=\;e^{-\alpha l}$$

Where $q_{0}^{"}$ , $q^{"}$ and α are incident heat flux, flux transmitted through the planer volume of depthl and absorption coefficient respectively.^[80]^ The interaction of light from xenon flash with precursor can be explained by combining laws of photophysics and photochemistry. The energy carried by the electromagnetic waves depends on the wavelength (frequency) given by Planck's law,

(4) E=hν

Where E,h and ν are energy of radiation, frequency of radiation, and Planck's constant respectively. On the interaction with molecules, this energy can be either excitation energy or bond dissociation energy per mole for the precursor molecules, which can be obtained by,

(5) $$E=Nh\nu =\frac{Nhc}{\lambda }=\frac{119627}{\lambda }\;kJ/mol\;$$

Where, c is speed of light ($=\;2.998\times 10^{8}{\;ms}^{-1}),\;N\;$ is Avogadro's number ($=\;6.022\times 10^{23}{\;mol}^{-1}\;),\;$ and lambda is wavelength of radiation. The wavelength range of the source (xenon flash lamp) light is from 200 nm to 1100 nm, which gives a range E from 108 kJ/mol to 598 kJ/mol.

**Figure 3:**
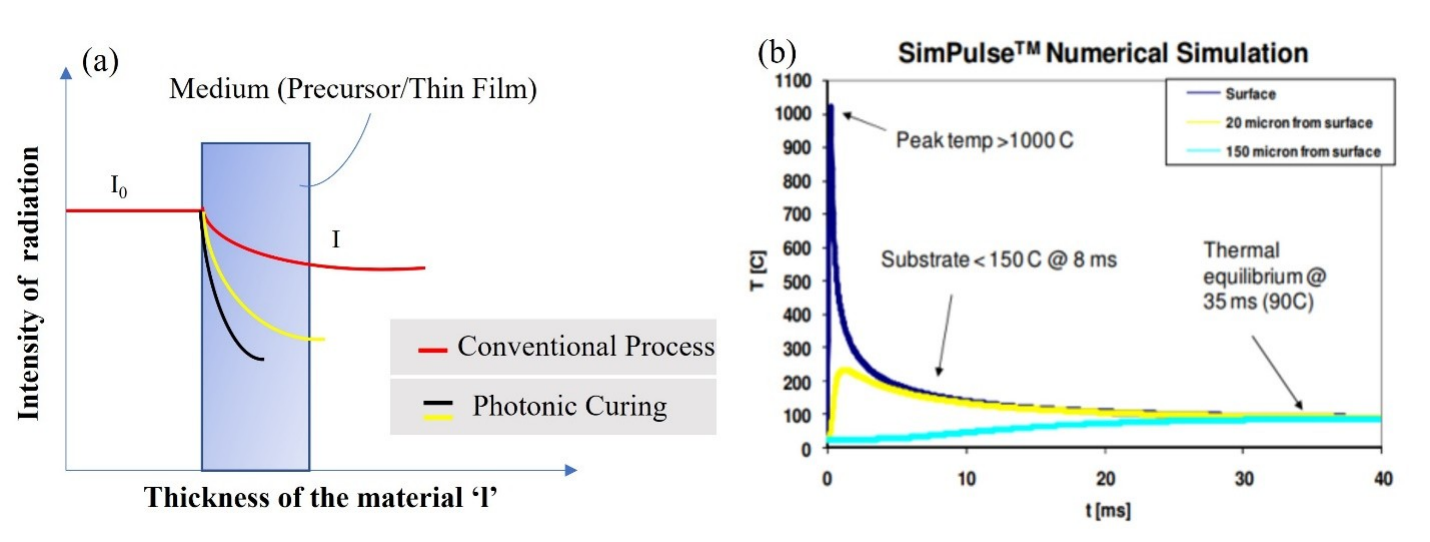
(a) Intensity of incident radiation vs. thickness of the material, the red line shows for the conventional thermal equilibrium processes the intensities varies by following the Beer‐Lambert law, I_0_ is initial intensity whereas I is the intensity at any instant of time (and thickness). (b) Temperature vs. time profile for substrate and thin film temperature during photonic curing showing peak temperature is higher than 1000 ^o^C but substrate remains relatively cool.

The photochemical reactions start on the absorption of radiation by the molecules as stated by the Grotthus‐Draper principle. The absorption of radiation obeys Beer‐Lambert Law.^[92]^ In Figure 3(a) we can see how the intensity of incident radiation changes across the medium. Beer‐Lambert law in general can be given by,

(6) $$I=I_{0}\;e^{-\alpha l}$$

**Figure 4:**
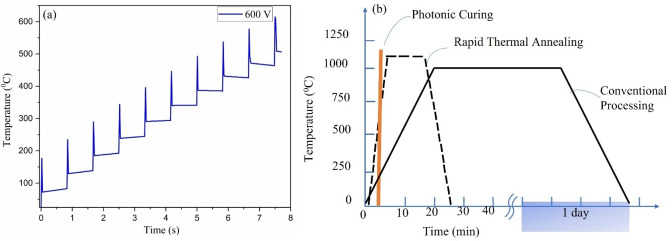
(a) Simulated temperature Vs. time profile for photonic curing of Ge and Si precursor thin films at 600 V bank voltage for 10 pulses, (b) Temperature Vs. time comparison for photonic curing (orange line), rapid thermal annealing (black dotted line) and conventional processing (black solid line).

WhereI is the intensity of radiation at the position l (l is zero at the surface of the material, and maximum at the bottom), $I_{0}\;$ is the initial intensity of the radiation alpha is the absorption coefficient. In case of photonic curing, the process is instantaneous, and it increases the temperature of the material up to 1000 °C (Figure (b))^[83]^ in a fraction of second. This will start photochemical reactions in the material and alpha will no longer be the same so we can say that α is alpha $\alpha \left(T\right)$ , where T is temperature. So, in that case, the intensity of radiation inside the material will no longer obey Beer‐Lambert law. During photonic curing, the changes in radiation intensity follows a trend as shown in Figure 3(a). Thus, the process is non‐equilibrium thermodynamic process. Since the time duration is very short in photonic curing that's why the heated precursor/thin film quenches very fast. To estimate the temperature of thin film during photonic curing, we used SimPulse; built‐in software for Pulse Forge 1300. The simulated temperature profile shows that temperature reaches ~600 ^o^C in during 10 pulses over the course of time (Figure 4a). However, this temperature profile cannot be taken as an accurate estimate because SimPulse software only considers thermal effects and does not incorporate photolytic effects.

Figure 4(b) shows the comparison of photonic curing with rapid thermal annealing and conventional processing. Photonic curing is an instantaneous process with a quenching rate ~10^6^ ^o^C/s, while quenching rates in rapid thermal annealing and conventional processing are generally ~3 ^o^C/s and~10^3^ °C/min, respectively.

During photonic curing, the pulsed light irradiation initiates photodecomposition and photothermal post‐treatment in material by providing electromagnetic and thermal energy.^[93]^ The photochemical reactions and photodecomposition will change the chemistry of the precursor depending on the temperature and other parameters such as precursor type, fluence, number of pulses, etc. But while the reactions will be happening and the precursor molecules will be in excited states, sudden quenching can often trap the molecules in metastable states before they reach an equilibrium state. Thus, photonic curing provides a route to trap metastable states of material with possibly having unique morphology and properties. Depending upon the energy imparted to precursor molecules, bond dissociation, nucleation and growth, the number density of the nanoparticles will vary.

**Figure 5:**
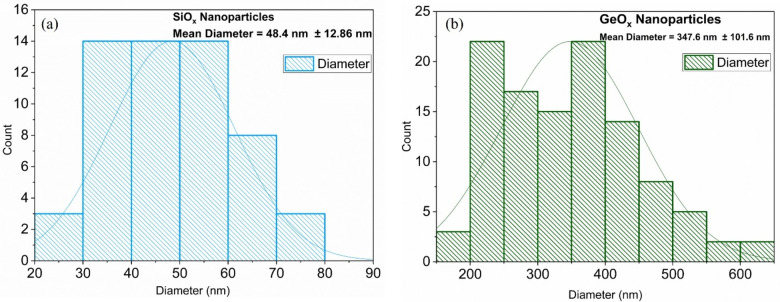
Histogram for (a) Si (b) Ge oxide nanoparticles, the histogram is obtained by using the SEM images shown in Figure 1. The average diameter of SiO_x_ and GeO_x_ nanoparticles is 48.4 nm and 347.6 nm respectively.

Figure 5 shows the histogram of nanoparticle distribution, SiO_x_ nanoparticles have less number density as compared to GeO_x_ nanoparticles. This difference in the number density of nanoparticles can be attributed to the volatility of Si precursor molecules. Due to the volatility, during spray coating, it doesn't adhere well to the substrate and as a result, less precursor remains on the substrate leading to fewer nanoparticles formation. Moreover, the molar mass of Si precursor (98.2 g/mol) is less than the molar mass of Ge precursor (252.9 g/mol).

**Figure 6:**
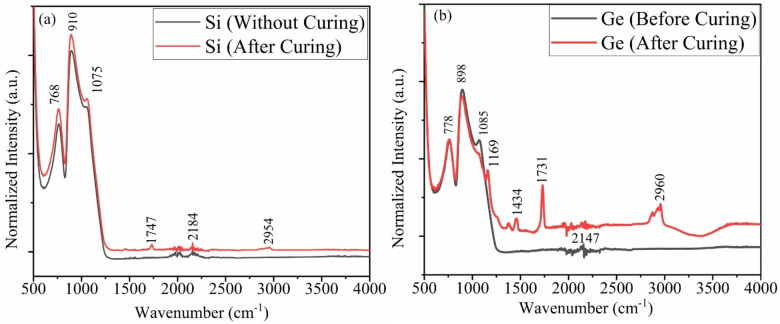
FTIR spectrum for (a) Si and (b) Ge precursors before and after curing. Various modes are labelled with corresponding wavenumbers. The peaks evolve with the change in oxygen content.

To further verify the synthesis of SiO_x_ and GeO_x_ nanoparticles, Fourier Transform Infrared Spectroscopy (FTIR) was performed using Nexus 670 ESP FTIR (Thermo Scientific, Waltham, MA). characterization was performed. The background consisting of 32 scans was collected immediately prior to each sample. The sample chamber was purged with nitrogen for 5 minutes before collecting data. For each sample, 64 repetitive scans were performed. Figure 6(a & b) shows FTIR results for as‐synthesized nanoparticles. In the fingerprint region (500 cm^−1^ to 1500 cm^−1^) of FTIR for GeO_x_ nanoparticles we can see a peak at 778 cm^−1^ which confirms the presence of oxygen‐containing germanium. The position of these peaks evolves with the oxygen content in the alloys. In particular, the stretching‐modes position of Ge−O−Ge bonds varies from 750 cm^−1^ to 870 cm^−1^, because of the peak's evolution with oxygen content, we can see a strong peak at 898 cm^−1^.^[94]^ The analysis of the functional group region (1500 cm^−1^ to 4000 cm^−1^) shows that the synthesized nanoparticles are ester functionalized due to the presence of a C=O stretching bond at 731 cm^−1^ (GeO_x_ nanoparticles) and 1747 cm^−1^ (for SiO_x_ nanoparticles), and C−C−O stretch bond at 1169 cm^−1^.[^95^, ^96^, ^97^] In FTIR spectra for Si the presence of peaks between 830 cm^−1^ to 1110 cm^−1^ shows the presence of strong Si−O bands.^[98]^ The peak at 2960 cm^−1^ in Ge and 2954 cm^−1^ in Si FTIR spectra indicates the presence of C−H bonds. It is observed that no silicon oxide peaks are observed in germanium oxide nanostructured films, this can be attributed towards the thickness of the germanium films which either blocked the infrared radiation to penetrate deep to the substrate or the signals received from glass substrate are very weak as compared to signals from germanium oxide nanostructure film.

**Figure 7:**
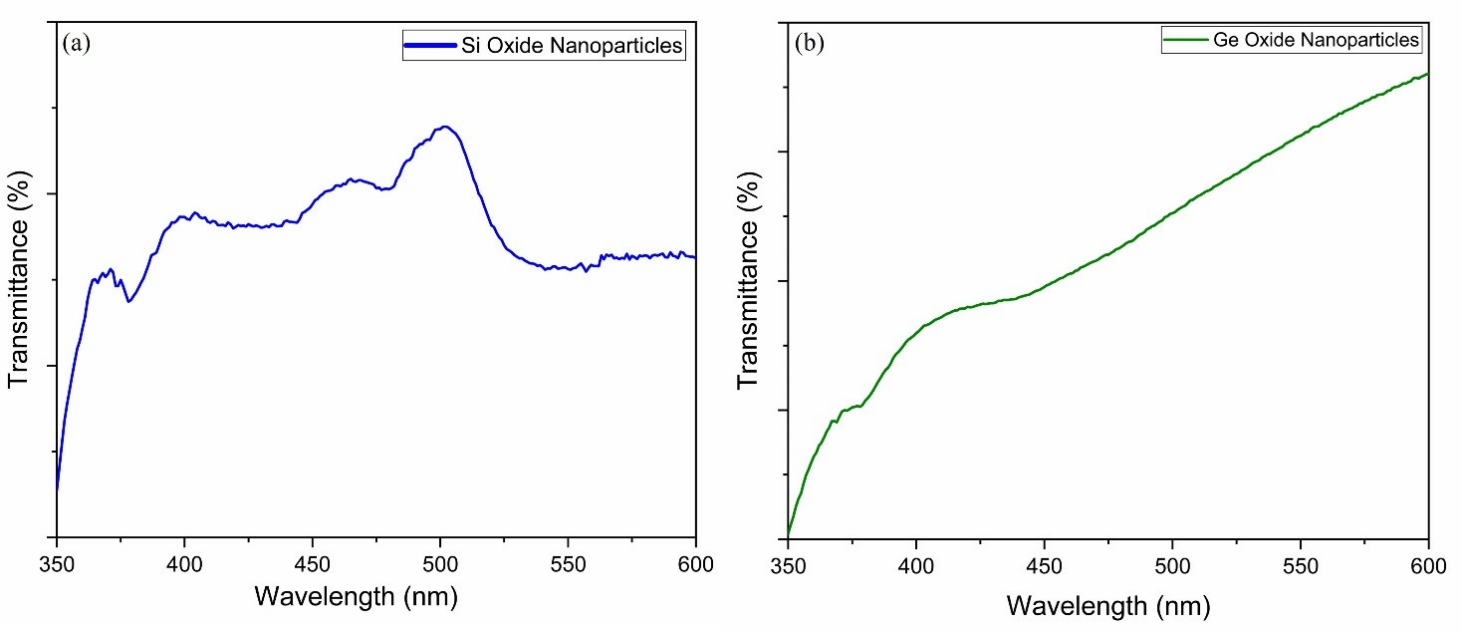
UV‐Vis results for (a) Si and (b) Ge oxide nanoparticles synthesized at xenon flashlamp bank voltage 600 V using 10 pulses. The transmission spectra shows maximum transmission ~510 nm and ~500 nm for silicon and germanium based nanoparticles.

Low fluence UV‐Vis transmission spectra of photonic cured Si and Ge precursor films were obtained by using PerkinElmer Lambda 750 S spectrometer. Figure 7(a & b) shows the UV‐Vis results for Si and Ge oxide nanoparticles, respectively. Glass substrate spectrum is used as reference spectra and is subtracted from all UV spectra. The UV spectrum shows that SiO_x_ nanoparticles have maximum transmittance at 510 nm wavelength. The GeO_x_ nanoparticles UV spectrum shows maximum transmittance at the 500 nm wavelength range. Though these are low fluence, the non‐zero values in this range ensure that we know *a priori* that there will be absorption of the front edge of the photonic pulse allow cooperative mechanisms to continue for the remainder of the pulse. It might seem that UV‐Vis is meaningless because the spectra are for products, but it is possible that the nucleation and growth of the nanoparticles from precursor could occur in the duration shorter than the pulse train of pulses. Thus, the absorption of wavelengths cooperates with the mechanism of changing precursor to final nanostructures for the remainder of the pulse.

**Figure 8:**
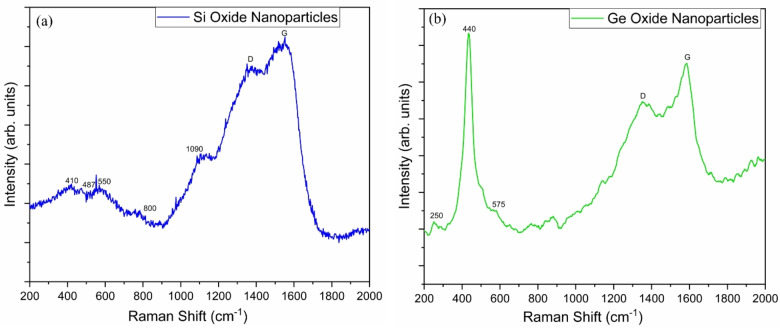
Raman results for (a) Si and (b) Ge oxide nanoparticles. The active Raman modes in both spectra indicate the presence of SiO_x_ and GeO_x_ nanoparticles while the D and G peaks show that the nanoparticles are not reduced and there is rGO.

Raman Spectroscopy was performed using DXR Raman Microscope (Thermo Scientific) with a 532 nm laser as the excitation source. Figure 8(a & b) shows Raman spectroscopy results of the synthesized nanoparticles. The Raman results for SiO_x_ presence of ~800 and 410–450 cm^−1^ bands which correspond to Si−O−Si symmetrical stretching and networking bending modes. The modes at 487 and 550 cm^−1^ are due to silicon oxide presence. The peaks at 1376 cm^−1^(D) and 1556 cm^−1^(G) show that silicon oxide nanoparticles are not reduced. The weak band at 1090 cm^−1^ is also present indicating the presence of silicon oxide.[^99^, ^100^, ^101^, ^102^, ^103^] Raman spectra for Ge oxide nanoparticles shows the presence and crystalline nature of Ge oxide nanoparticles. The band at 575 cm^−1^ is Raman actives modes (first order and second order) transverse optical phonon modes (TO and 2TO) are present which are TO modes of crystalline Ge. The three modes in the range of 250 cm^−1^ to 440 cm^−1^ are characteristic Raman active modes for GeO_2_.^[104]^ Same as the silicon oxide nanoparticle Raman Spectra, the peaks at 1376 cm^−1^(D) and 1556 cm^−1^(G) show that germanium oxide nanoparticles are not reduced.

**Figure 9:**
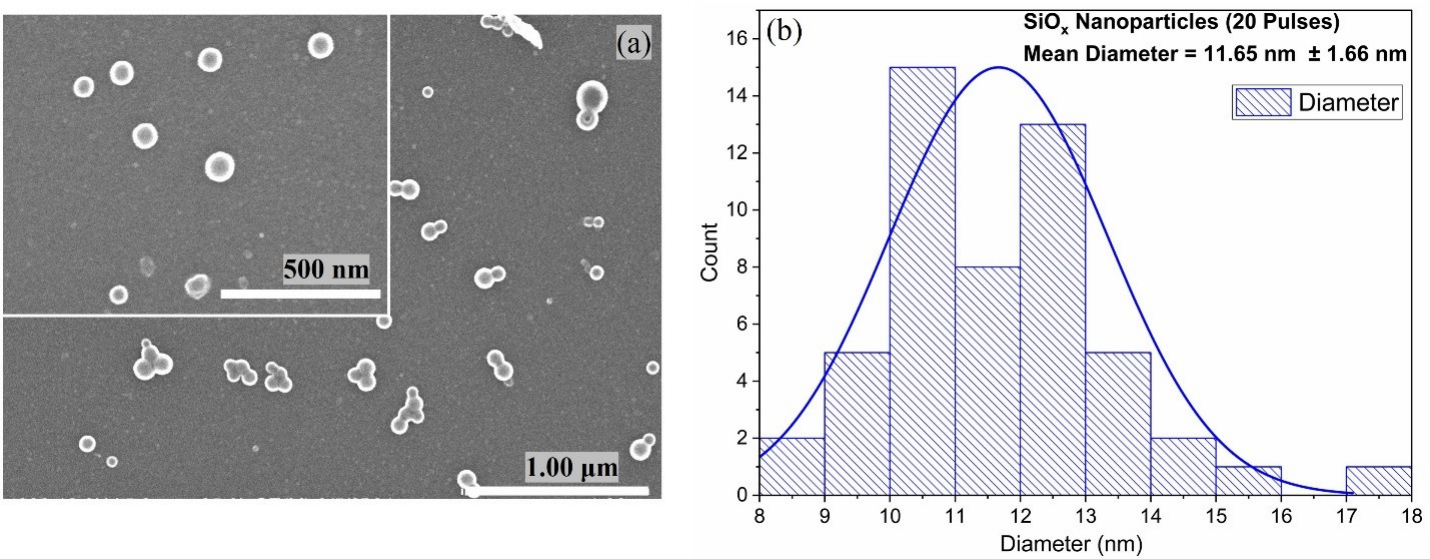
(a) SEM images for Si precursor photonically cured at 600 V for 20 pulses with high‐resolution SEM in the inset on the top left corner, (b) Histogram for the silicon oxide nanoparticles synthesized by photonic (10 pulses). The number density of nanoparticles decreased with increasing the number of pulses as well the size of nanoparticles also decreased.

Figure 9(a) shows SEM results for photonically cured Si precursor thin film for 20 pulses (keeping other PulseForge parameters the same). Comparison of Figure 1(a, b) and Figure 9(a) shows that if a thin film of precursor is coated by increasing number of pulses and hence total fluence, there will be a smaller number of particles per cm (Figure 9(a)), and chain‐like structure will also break up. The chances of agglomeration in the case of nanoparticles synthesized at high fluence during photonic curing since the nanoparticles are farther apart.

The size distribution of SiO_x_ nanoparticles is shown in Figure 9(b). The average size is 11.65 nm which is much less as compared to the SiO_x_ nanoparticles size (48.4 nm) synthesized by 10 pulses of photonic curing. Increasing the number of pulses increases the total fluence during photonic curing. At 10 pulses total fluence was 80 Jcm^−2^ while at 20 pulses total fluence is 160 Jcm^−2^. This results in more energy imparting to the thin film resulting in a direct effect on nanoparticles size. The decrease in nanoparticle size is due to achievement of an equilibrium composition of surface versus bulk volume of nanoparticles. This decrease in nanoparticle size shows the rapidness; that how quickly nanostructures are quenched, and that speed is even more quick at higher temperatures/fluence than at lower temperatures/fluence.

## Conclusions

Photonic curing of Si and Ge precursors in current studies opened the possibility of developing novel, economic and industrial‐scale techniques for nanoparticle synthesis. We synthesized silicon and germanium oxide nanoparticles at bank voltage 600 V for 10 pulses and extended the study to observe the effect of number of pulses on nanoparticle synthesis. The study showed that increasing the number of pulses results in small size and less density non‐agglomerated nanoparticles. There are other possible parameters of PulseForge that we suggest varying to study the effect on nanomaterials synthesis. The freedom of tuning the size and morphology of nanoparticles in a reproducible manner by adjusting the parameters of PulseForge (voltage, fluence, and pulse train) makes it a promising synthesis method for controlled synthesis of nanoparticles which is still a continuous struggle in the field of nanofabrication. However, there are quite a few challenges to utilizing this technique for industrial‐scale synthesis of nanoparticles, which includes the yield of nanoparticles and understanding the mechanism of the process. Our work presented will help to understand the process during photonic curing and eventually, a fundamental step to utilize photonic curing for large‐scale synthesis of Si and Ge oxide nanoparticles for lithium‐ion batteries. Further control in properties of nanoparticle could be achieved by using higher molarity of precursor, identifying better precursors with computational studies, and changing the processing environment during photonic curing. Tuning the processing parameters as well as processing in a reducing atmosphere of forming gas or vacuum could provide more possibilities.

## Experimental Section

### Sample preparation

Silicon precursor, trimethylsilylacetylene 98 %, and germanium precursor Germanium (IV) ethoxide 99.5 %, were purchased from Sigma Aldrich. ACS Reagent Grade Acetone (99.9 %) was purchased from Sycamore Life Sciences to use as solvent. Three step cleaning protocol was followed to clean the glass substrates. For glassware and substrate cleaning, ethyl alcohol, acetone, and 18 MΩ de‐ionized (DI) water were used as cleaning solvents. The cleaning procedure consisted of 10 minutes sonication in acetone followed by 10 minutes sonication in ethyl alcohol and DI water. The molarity of precursor solutions was kept 0.1 M. Air‐spray (Paasche Airbrush, Chicago, IL, USA) was utilized to deposit thin films on substrates with nitrogen gas (2 bar) under ambient conditions. Due to the high evaporation rate of acetone, the precursor droplets landing on the substrate kept 15 cm away from the spray gun dries out instantly and makes thin precursor films.

### Photonic Curing

PulseForge 1300, NovaCentrix Corp., Austin, TX, USA was used to perform photonic curing of as‐prepared thin films of Si and Ge precursors. As‐prepared thin film substrate was placed on 1.5 mm thick fused silica to prevent the heating of the substrate from the aluminum stage. The distance between the lamp and the aluminum stage is kept at 10 mm. The lamp voltage was set to 700 V with a fire rate of 1.2 Hz, and 3200 μs envelope. The photonic curing was performed in the ambient atmosphere with a total fluence of 80 Jcm^−2^ by dividing it over 10 pulses (8 Jcm^−2^ fluence per pulse). The total fluence was changed from 80 Jcm^−2^ to 160 Jcm^−2^ by changing the number of pulses from 10 to 20. Fluence for each trial was measured through in situ bolometer.

## Conflict of interests

The authors declare no conflict of interest.
